# Molecular and histopathological characterization of *Ascaridia galli* and *Eimeria tenella* co-infection in *Numida meleagris*

**DOI:** 10.3389/fvets.2025.1622170

**Published:** 2025-09-26

**Authors:** Marwa M. Attia, Mahmoud A. Mahmoud, Mohamed Abdelsalam, Layla A. Almutairi, Mohammed A. Alqahtani, Sultan Mohammed Areshi, Mohamed T. El-Saadony, Khaled A. El-Tarabily, Heba M. Salem

**Affiliations:** ^1^Department of Parasitology, Faculty of Veterinary Medicine, Cairo University, Giza, Egypt; ^2^Department of Pathology, Faculty of Veterinary Medicine, Cairo University, Giza, Egypt; ^3^Department of Aquatic Animal Medicine and Management, Faculty of Veterinary Medicine, Cairo University, Giza, Egypt; ^4^Department of Biology, College of Science, Princess Nourah bint Abdulrahman University, Riyadh, Saudi Arabia; ^5^Department of Biology, College of Science, King Khalid University, Abha, Saudi Arabia; ^6^Department of Biology, College of Science, Jazan University, Jazan, Saudi Arabia; ^7^Department of Agricultural Microbiology, Faculty of Agriculture, Zagazig University, Zagazig, Egypt; ^8^Department of Biology, College of Science, United Arab Emirates University, Al Ain, United Arab Emirates; ^9^Department of Poultry Diseases, Faculty of Veterinary Medicine, Cairo University, Giza, Egypt; ^10^Department of Diseases of Birds, Rabbits, Fish and their Care and Wildlife, School of Veterinary Medicine, Badr University in Cairo (BUC), Cairo, Egypt

**Keywords:** clinical examination, helmeted guineafowl, molecular surveillance, parasitological analysis, poultry parasites, phylogenetic analysis

## Abstract

**Introduction:**

The helmeted guineafowl is a ground-dwelling bird native to Africa, easily recognized by its bald, bluish-gray head and the distinctive horn-like casque (helmet) on top of its head. Parasitic coinfection with *Ascaridia* worms and *Eimeria* in chickens poses a significant health challenge, as both parasites damage the intestinal tract and impair nutrient absorption. *Ascaridia galli* competes for nutrients and causes mechanical irritation, while *Eimeria tenella* induces mucosal injury and inflammation. Their combined effect leads to severe enteritis, reduced growth performance, poor feed conversion, and increased susceptibility to secondary infections. This synergistic impact exacerbates economic losses in poultry production and highlights the importance of integrated parasite control strategies.

**Methods:**

This study investigated the cause of mortality in helmeted guineafowl on a private farm. Clinical examination, necropsy, parasitological analysis, molecular characterization, and histopathological examination were conducted.

**Results:**

Preliminary findings indicated a mixed gastrointestinal parasitic infection, with *A. galli* and *E. tenella* identified as the causative agents of co-infection. Molecular analysis targeting the ITS rDNA and COX1 regions of *A. galli* and the ITS and 18S rDNA regions of *E. tenella* confirmed their identities and revealed genetic diversity among the isolates. Phylogenetic analysis clustered the isolates within well-supported clades of their respective species. Clinical signs included depression and sporadic hemorrhagic droppings, while postmortem lesions varied, featuring enteritis, hemorrhagic typhlitis, splenic necrosis, and hepatic lesions. Histopathological examination revealed severe intestinal damage, including hemorrhage, epithelial desquamation, and the presence of multiple parasite developmental stages. The co-infection led to a 10% mortality rate.

**Discussion:**

The current study offers insights into the impact of *A. galli* and *E. tenella* co-infection in helmeted guineafowl, underscoring the importance of molecular surveillance in monitoring poultry parasite populations. Additional research is recommended to establish routine parasitological monitoring, implement targeted deworming initiatives, enhance sanitation, and enforce biosecurity protocols to reduce parasite load and prevent epidemics.

## Introduction

Helmeted guineafowl (*Numida meleagris*) are birds native to sub-Saharan Africa but introduced in various regions around the globe for domestic rearing and/or production. These birds are deemed more resistant to parasitic infections (1). Poultry production faces significant challenges from parasitic infections, with more than 30 helminths identified in hens (2). Among these, *Ascaridia galli* stands out as the most prevalent, often occurring in mixed infections with the caecal nematode *Heterakis gallinarum* (3–5).

While *A. galli* has been observed in chickens across various housing systems globally, its prevalence was lower in laying hens housed in traditional battery cages (6, 7). The persistence of *A. galli* in poultry environments is facilitated by its resilient eggs, which can survive for extended periods. Although most ascarid eggs are destroyed within months, a small percentage (up to 3%) can remain viable for up to 2 years (8). This longevity, coupled with the parasite’s high reproductive rate and the host’s compromised immune response, leads to an escalating intensity of infection over time (9). As the largest intestinal helminth in chickens, *A. galli* significantly impacts egg and meat production, thereby affecting both economic outcomes and animal protein availability (10–12).

The parasite’s life cycle is direct, involving a single host. The adult worms in the small intestine produce eggs that are expelled into the environment (13). These eggs are protected by three layers: an outer thin albuminous layer, a thick, resistant covering, and an inner permeable vitelline membrane (2, 13). The eggs develop internally to the infectious third larval stage (L3) without hatching in the environment (14). Under typical conditions in chicken barns, *A. galli* eggs rapidly develop into infectious larvae, a process known as embryonation in ovo (14, 15). Laboratory studies have demonstrated that under optimal conditions, nearly 88% of *A. galli* eggs complete development within 1–2 weeks (16, 17).

The pathogenesis of *A. galli* infection primarily stems from the larval stages, although both adult and immature parasites contribute to intestinal health deterioration (18). Transmission of *A. galli* occurs through ingestion of contaminated food or water or through ingestion of earthworms containing larvae (15). Earthworms can serve as paratenic hosts, ingesting the infectious stage and subsequently transmitting the infection to definitive hosts (17). Infection via earthworm consumption is more efficient than direct egg ingestion from the environment, provided the earthworm is consumed within 96 h of ingesting the eggs (17).

In addition to helminth infections, the poultry industry faces significant challenges from protozoan diseases, particularly coccidiosis. This disease accounts for 6–10% of all broiler mortalities and causes substantial economic losses worldwide (19). Coccidiosis is caused by apicomplexan protozoa of the genus *Eimeria*, affecting both domestic and wild chickens (20). The severity of coccidiosis can be classified into three levels: coccidiosis (a mild infection with no adverse effects), subclinical coccidiosis, and clinical coccidiosis (21–24).

Subclinical infections can reduce bird growth performance through various mechanisms, including reduced voluntary feed intake, decreased nutrient digestibility, and diversion of nutrients for tissue repair and immune responses (25–27). The impact of *Eimeria* infections on bird growth performance is influenced by factors related to the pathogen, host, and environment (28). Host factors such as age, sex, genetic line, and pathogen factors like *Eimeria* species can modulate the impact of the infection. The disease progression, including incubation, acute, and recovery phases, is crucial in determining the overall effect on performance (29).

Multiple *Eimeria* species affect chickens, each with its specific predilection site in the gut, oocyst morphometrics, and immunogenicity (30). Reid et al. (31) classified *Eimeria* species based on their effects on the host: hemorrhagic (*E. tenella*, *E. necatrix*, and *E. brunetti*), malabsorptive (*E. maxima* and *E. acervulina*), and lesser degree infections (*E. mitis* and *E. praecox*). Four species are of particular concern due to their global disease and economic impact: *E. acervulina, E. maxima, E. tenella*, and *E. necatrix*. The life cycle of *Eimeria* spp., involves both exogenous and endogenous stages, with well-documented durations that vary slightly between species (31). For instance, the prepatent (incubation) phase ranges from 4 days for *E. acervulina* to 5–6 days for *E. maxima* and 6–7 days for *E. tenella* (32).

The endogenous phases (schizogony and gametogony) disrupt host intestinal cells to form parasite “zoites.” Coccidiosis typically manifests as an acute invasion and destruction of intestinal mucosa by the protozoa *Eimeria* or *Isospora*. Clinical symptoms include diarrhea, fever, decreased appetite, weight loss, and emaciation, with severe cases potentially leading to death (33). The disease compromises the mucous membranes throughout the intestinal tract, resulting in absorption imbalances that cause diarrhea and, in extreme cases, mortality (34). However, many infections remain subclinical, posing challenges for detection and management (34).

Molecular sequencing techniques have revolutionized the identification and characterization of parasites in birds, offering unprecedented accuracy and reliability in diagnosing infections such as those caused by *A. galli* and *E. tenella* (35–37). These advanced methods enable precise species identification and provide valuable insights into the genetic diversity of parasites, contributing significantly to our understanding of host–parasite dynamics and the epidemiology of avian diseases in natural ecosystems (37).

Interestingly, *A. galli* and *Eimeria* spp., often occur as concurrent infections in poultry with prominent pathological lesions potentially exacerbating the negative impacts on host health and productivity (38–40). This co-occurrence can lead to complex interactions between the parasites and the host immune system, further complicating diagnosis and treatment strategies. Furthermore, co-infections’ timing can significantly influence the severity of the disease and the induced pathological lesions, with *A. galli* infection preceding other etiological agents, resulting in more severe outcomes (40, 41).

Given the significant impact of parasitic infections on poultry health and production, there is a critical need for further investigation into the interactions between *A. galli* and *Eimeria* spp. in concurrent infections. Thus, the current study aimed to investigate the causative agents of mortality in black-colored chickens (*N. meleagris*) on a private farm, with a particular focus on identifying and characterizing the parasitic infections present. The study also aims to molecularly characterize both parasites using COX1, ITS, and 18S rRNA sequencing and to assess the impact of concurrent infections through histopathological examination.

## Materials and methods

### Ethical approval

The work was carried out in accordance with the IACUC guidelines and was approved by the Ethical Committee of the Faculty of Veterinary Medicine, Cairo University, Cairo, Egypt, with the code “Vet CU 8032022511.”

### Study design and sample collection

The current study was conducted in response to an unusual mortality pattern observed among helmeted guineafowl on a private farm. The farm reported a significant increase in chicken mortalities over a two-week period, reaching approximately 30%. This mortality rate initially indicated the overall expected losses among the entire flock on the farm over the 2 weeks preceding our examination, as conveyed by the farm owner, which prompted an inquiry into suspected causal agents, notably parasitic diseases.

Following the initial report, a series of farm visits were conducted between October to November 2024. During these visits, comprehensive farm inspections were carried out, focusing on housing conditions, feed quality, and general management practices that might contribute to disease susceptibility.

A total of 60 chickens were included in the study, and this number of birds was the total number of the remaining birds after mortality and they were clinically diseased birds and available for monitoring and intervention during our visits. The mortality during the farm investigation has reached 10% (6 birds out of 60); the age of dead birds ranges from 18 to 30 months. Birds were selected based on clinical signs suggestive of parasitic infection, including diarrhea, weight loss, and reduced egg production.

Sampling included freshly dead birds as well as droppings samples from the surrounding environment. The samples were then transported to the laboratory of the Department of Parasitology, Faculty of Veterinary Medicine, Cairo University, Giza, Egypt, for comprehensive post-mortem examination and tissue sampling under sterile conditions. Fecal samples were collected in sterile containers with 2.5% potassium dichromate solution for oocyst preservation.

### Clinical examination and necropsy

Diseased birds were subjected to thorough clinical examination, recording all observable symptoms. Birds that succumbed to the disease were promptly necropsied following standard veterinary protocols recommended by Berhe et al. (42). During necropsy, all visceral organs were systematically examined for gross pathological changes.

Particular attention was paid to the gastrointestinal tract, with the mucosa of the duodenum, jejunum, ileum, and ceca being carefully inspected for the presence of parasites, especially *Eimeria* and *Ascaridia* spp., following the method described by Razmi and Kalideri (43).

### Parasitological examination

#### Microscopic examination

Wet smears of mucosal scrapings from intestinal and caecal tissues were prepared and examined using Olympus BH-2 (Olympus Optical Co. Ltd., Tokyo, Japan) light microscope equipped with a digital camera and software (Jenoptik ProgRes Camera, C12plus, Frankfurt, Germany). *Eimeria* spp. were identified based on the site of infection, oocyst morphology (including size, color, presence or absence of micropyle and micropyle cap), and sporulation time, according to the criteria established by Long and Rose (44).

#### Oocyst isolation and quantification

*Eimeria* spp. oocysts were isolated from caecal and lower intestinal mucosa using the saturated sodium chloride flotation technique as described by Permin and Hansen (45). Oocyst quantification was performed using a modified McMaster’s oocyst-counting technique (46, 47). For sporulation, intestinal contents were collected from freshly dead birds and incubated in a 2.5% aqueous solution of potassium dichromate at 27 °C for 48–72 h with regular aeration.

#### Helminth collection and preservation

Helminths were carefully extracted from the intestinal tract and washed repeatedly with phosphate-buffered saline (PBS, pH 7.2). Specimens were preserved in 70% glycerine alcohol for morphological studies and mounted using glycerol jelly, following the method of Salem and Attia (48).

### Molecular identification of *Ascaridia* sp.

Individual worm specimens underwent thorough cleansing with five sterile distilled water rinses before genomic DNA extraction using a DNeasy tissue kit (Qiagen, Hilden, Germany) following manufacturer protocols. DNA quality and concentration were assessed using a NanoDrop ND-1000 spectrophotometer (Thermo Fisher Scientific Inc., Waltham, Massachusetts, United States).

Two complementary genetic regions were targeted to ensure robust species identification and phylogenetic resolution. These were the internal transcribed spacer (ITS) region of ribosomal DNA and the cytochrome c oxidase subunit 1 (COX1) gene. The ITS region was amplified using universal primers BD1 (5′-GTCGTAACAAGGTTTCCGTA-3′) and BD2 (5′-TATGCTTAAATTCAGCGGGT-3′) (49), while COX1 amplification employed nematode-specific primers Ag Cox1F (5′-ATTATTACTGCTCATGCTATTTTGATG-3′) and Ag Cox1R (5′-CAAAACAAATGTTGATAAATCAAAGG-3′) (37).

PCR reactions were optimized in 50 μL volumes containing 5 μL of 10 × buffer, 5 μL of each dNTP at 10 mM, 10 μL of each primer at 1 pmol/μl, 0.3 μL of Taq polymerase (5 U/mL), 2.5 μL magnesium chloride (50 mM), and 2 μL of extracted gDNA. Amplification products were visualized on 1.5% agarose gels in 1X TAE buffer and purified using QIAquick PCR Purification Kit (Qiagen) prior to bi-directional Sanger sequencing (Macrogen Inc., Seoul, South Korea).

#### Phylogenetic analysis

Consensus sequences were aligned with representative GenBank sequences spanning *Ascaridia* spp., *Heterakis* spp., *Ascaris* spp., and related ascaridoid taxa using ClustalW for multiple sequence alignment in MEGA 11 (50). Outgroup selection was based on phylogenetic distance and data availability with *Mastophorus muris* (MK829006) for ITS analysis and *Neoentomelas asatoi* (LC632117) for COX1, representing closely related but distinct nematode lineages.

Maximum Likelihood phylogenetic reconstruction employed the GTR + G + I model, selected for its ability to accommodate variable substitution rates and site-specific evolutionary constraints common in ribosomal and mitochondrial sequences. Bootstrap support (1,000 replicates) provided statistical validation of tree topology.

### Molecular identification of *Eimeria* sp.

Purified oocysts underwent four freeze–thaw cycles to disrupt oocyst walls before single-oocyst DNA extraction using DNeasy Tissue Kit (Qiagen). The complementary marker approach targeted both 18S rDNA and ITS regions to maximize phylogenetic resolution and enable comparison with existing databases. The 18S rDNA was amplified using primers ERIB1 (5′-ACCTGGTTGATCCTGCCAG-3′) and ERIB10 (5′-CTTCCGCAGGTTCACCTACGG-3′), while the ITS1-ITS2 regions were targeted using primers ITS-1 (5′-GGATGCAAAAGTCGTAACACGG-3′) and ITS-2 (5′-TCCTCCGCTTAATAATATGC-3′) (35). Both primer sets were selected for their proven efficacy in identifying apicomplexan parasites.

Phylogenetic analysis followed identical protocols, using appropriate outgroups: *Coccidia* sp. (MH590232) for ITS analysis and *Isospora belli* (AF106935) for 18S rDNA analysis. These outgroups were chosen to represent related coccidian parasites while maintaining sufficient evolutionary distance for tree rooting.

### Histopathological examination

The histopathological examination involved tissue samples from the duodenum, ileum, and cecum. The samples were preserved in 10% neutral buffered formalin for 24 h, then routinely processed, embedded in paraffin, sectioned at 3–5 μm thickness, and finally stained with hematoxylin and eosin using standard protocols (51).

Histopathological lesions and the presence or absence of *Eimeria* spp. and *Ascaridias* were recorded. Tissue sections were examined, and the lesions were photographed using an Olympus light microscope.

## Results

### Clinical examination and postmortem findings

**Figure 1:**
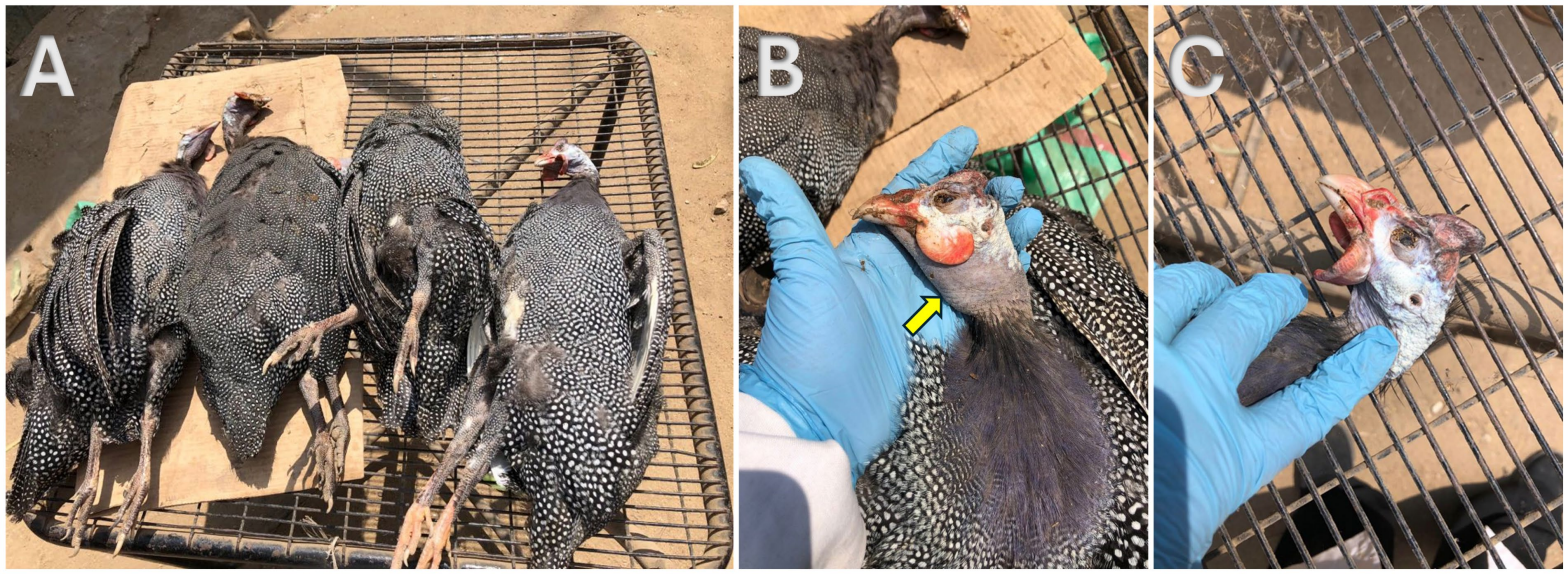
Postmortem examination of freshly dead helmeted guineafowl showing **(A)** high mortality during the farm investigation; **(B)** submandibular edema (yellow arrow); **(C)** planes in comb and wattles.

The investigated birds exhibited a range of clinical signs indicative of severe parasitic infection. As seen in Figure 1, the most common signs observed were mortalities, ruffled feathers, lack of appetite, drowsiness, planes in com and wattles, inability to walk, some birds revealed submandibular oedema, many birds were seen huddling together, and a significant proportion displayed bloody diarrhea. Out of the 60 birds examined, 10% succumbed to the infection during the study period.

**Figure 2:**
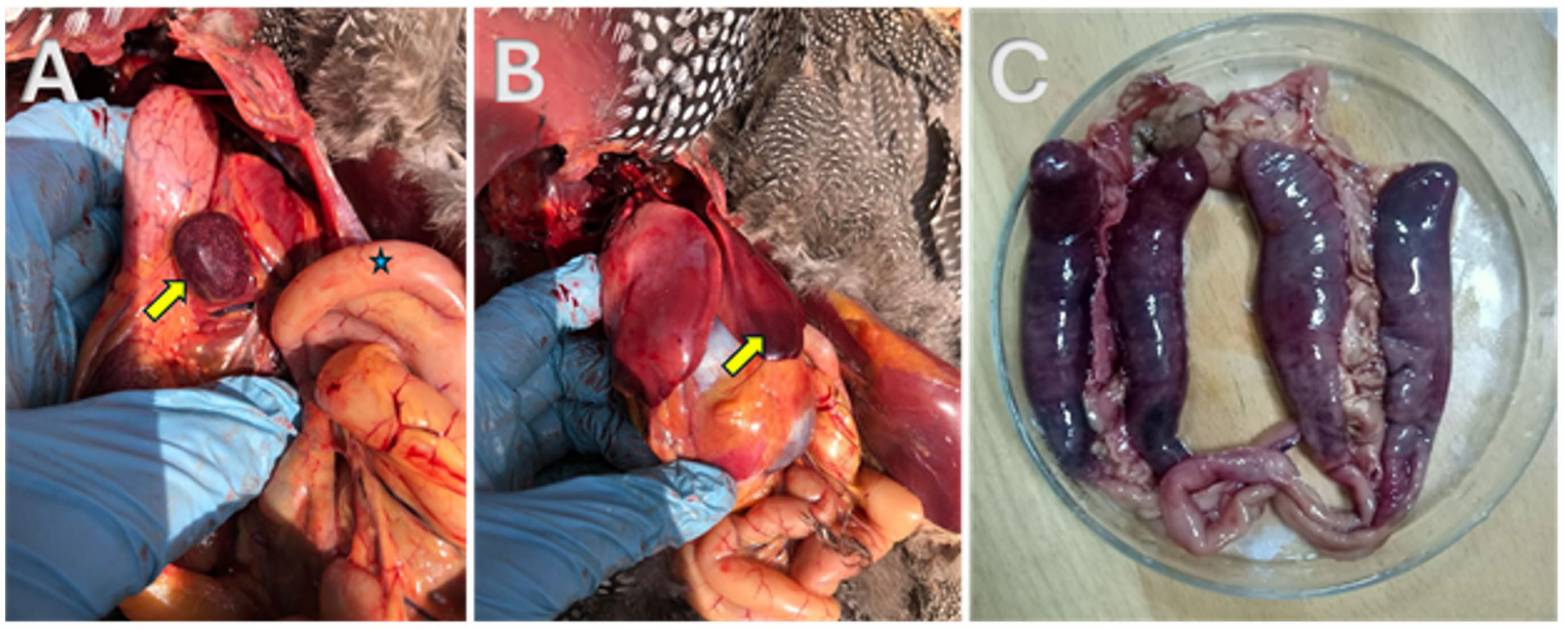
Postmortem examination of freshly dead helmeted guineafowl showing **(A)** necrosis in the spleen (yellow arrow); **(B)** subcapsular hemorrhage in the liver (yellow arrow); **(C)** blood sausage appearance of the two blind ceci.

As seen in Figure 2, freshly dead birds’ postmortem examinations showed consistent, recognizable lesions as the caeca’s appeared filled with blood; sausage-like appearance and the mesenteric blood vessels’ engorgement were the most common findings. The ceca had bloody contents when it was cut, and in a few cases, cecal cores were visible. The appearance of the infected hens’ spleens varied; some were pale, while others had surface petechial hemorrhages with necrosis. Areas of mucosal thickening and hyperemia were frequently observed in the small intestine, especially the ileum. The liver showed multiple pale areas with subcapsular hemorrhages.

### Morphology and parasitological examination

The parasites identified in this study were *A. galli* and *E. tenella*. Fecal flotation tests revealed the presence of *Eimeria* oocysts in 90% of examined samples, with an average oocyst count of 8,000–12,000 ± 100 oocysts per gram of feces. *A. galli* eggs were detected in 60% of fecal samples, with an average egg count of 600–1,200 ± 25 eggs per gram of feces.

**Figure 3:**
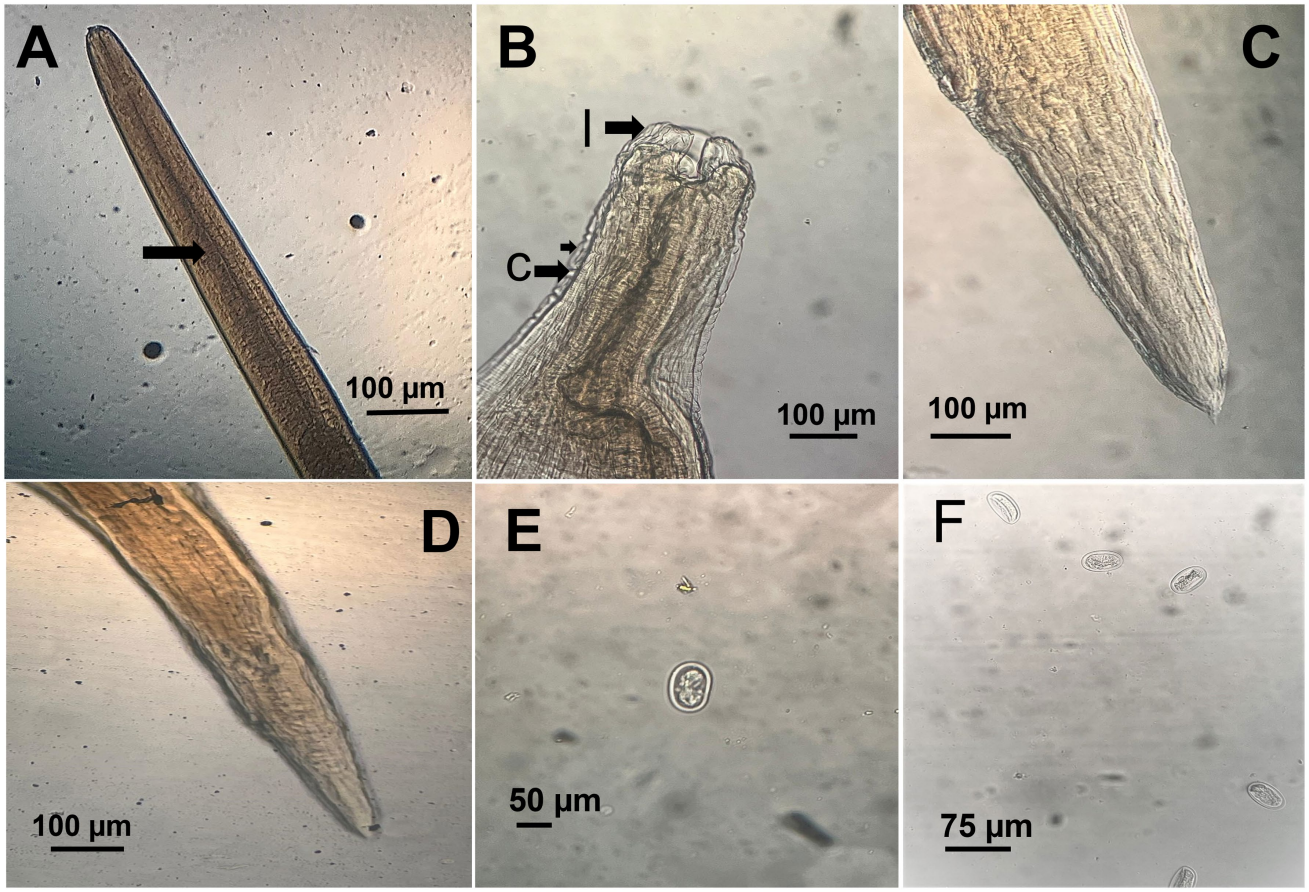
Microscopic morphology of *Ascaridia galli*. **(A)** Anterior part of adult worm showing the thick cuticle (arrow). **(B)** Close view of the cephalic end displaying trilobed lips (l) and cervical alae (c). **(C)** Posterior end of male with characteristic pointed tail. **(D)** Posterior end of female with bluntly rounded tail. **(E)** Unembryonated *A. galli* egg recovered from feces, showing smooth shell. **(F)** Multiple *A. galli* eggs in flotation preparation.

*A. galli* specimens exhibited distinct sexual dimorphism. Female worms, ranging from 75 to 115 mm in length, were notably larger than males. The body was cylindrical, creamy-white, and semi-transparent, with a thick, proteinaceous cuticle displaying transverse striations. The anterior end was characterized by three large, trilobed lips encircling a large mouth. Male *A. galli*, measuring between 50 and 76 mm in length, had distinctly pointed and curled tails with 10 pairs of caudal papillae (Figure 3).

**Figure 4:**
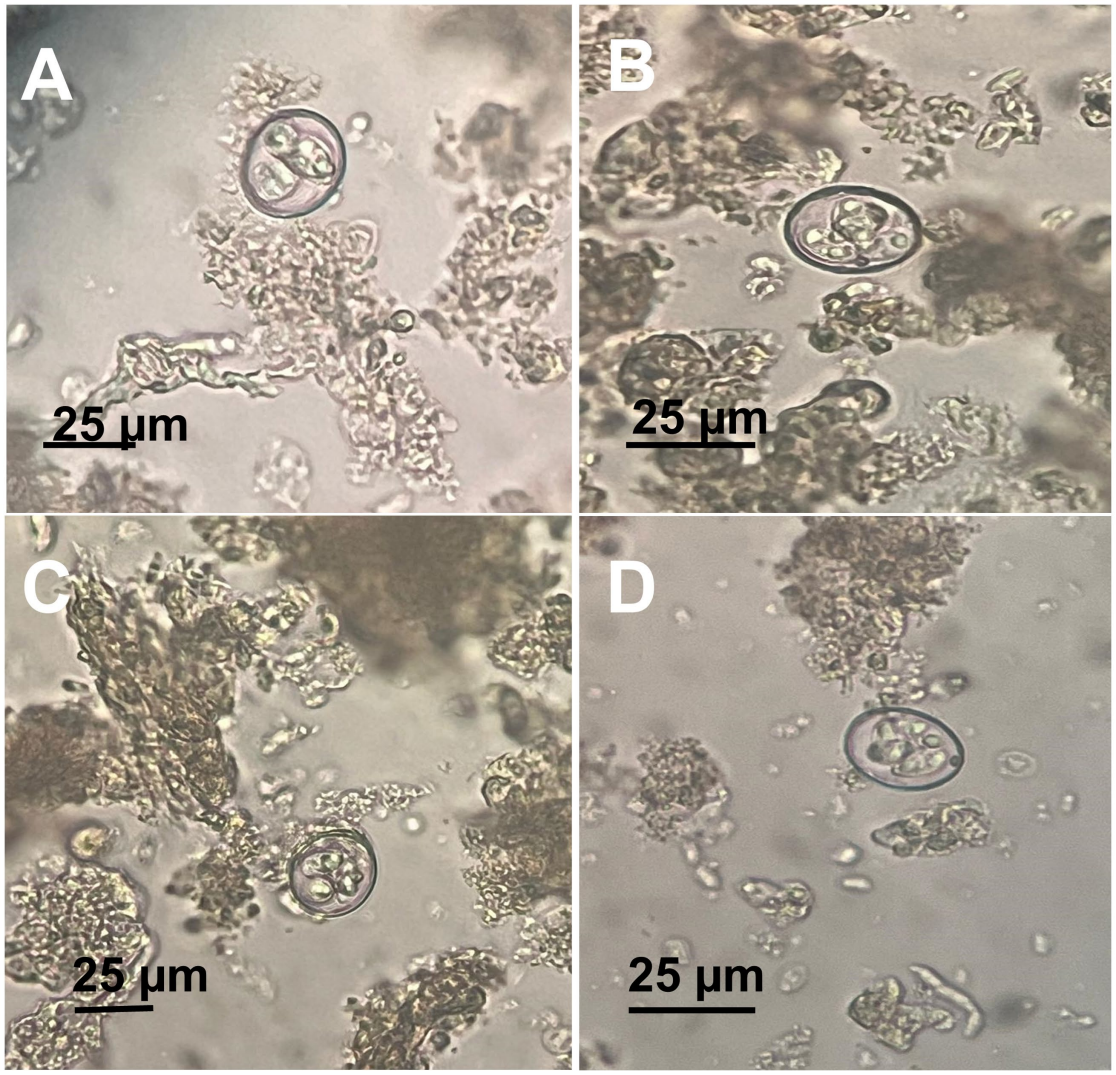
Microscopic appearance of *Eimeria tenella* sporulated oocysts. **(A–D)** Different views showing a fully mature sporulated oocyst.

Eggs collected from infected birds’ feces were oval shaped with smooth shells, measuring 75–96 μm by 48–59 μm. Adult *A. galli* worms were recovered from the small intestines of half of the necropsied birds, with an average worm burden of 15–30 worms per bird. On the other hand, *E. tenella* oocysts were identified by their ovoid shape and double-layered oocyst wall, lacking a micropyle and micropylar cap, sporulated oocysts measured 25 (21–27) μm in length and 18 (15–29) μm in width. Direct microscopic examination of intestinal scrapings revealed various developmental stages of *Eimeria*, including schizonts, merozoites, and gametocytes, in the majority of examined birds (Figure 4).

### Molecular identification of *Ascaridia* sp.

#### ITS and COX1 sequencing of *Ascaridia* sp.

Table 1 demonstrates that the ITS rDNA region was effectively amplified from three distinct *Ascaridia* specimens, producing sequences of 981, 982, and 972 base pairs, with accession numbers PQ047113, PQ047114, and PQ047115, respectively. The COX1 gene was successfully amplified from the same three specimens, producing sequences of 526, 525, and 526 base pairs, deposited as PQ106343, PQ106344, and PQ106345, respectively. Sequence analysis confirmed taxonomic identification as *A. galli*.

**Table 1:** Summary of *Ascaridia galli* molecular characterization.

Marker	Length (bp)	Accession number	Intraspecific similarity (this study)	Similarity other *A. galli*	Interspecific similarity range
ITS	972–982	PQ047113–PQ047115	98.67–98.98%	97.26–99.08%	*A. columbae* (95.20–96.78%)
COX1	525–526	PQ106343–PQ106345	98.67–99.24%	96.96–99.62%	*A. columbae* (85.52%)

Detailed comparison revealed specific intraspecific variations: ITS sequence PQ047113 differed from PQ047114 by 9 base pair substitutions and 1 gap, while exhibiting 4 base pair differences and 9 gaps compared to PQ047115. COX1 sequence PQ106343 exhibited 7 base pair substitutions compared to PQ106344, and 4 base pair differences relative to PQ106345. Interspecific comparisons revealed lower similarities: *A. columbae* (95.20–96.78% for ITS, 85.52% for COX1), other *Ascaridia* species (76.53–87.94%), and *Heterakis* species (77.61–86.82%).

#### Phylogenetic analysis of *Ascaridia* sp.

**Figure 5:**
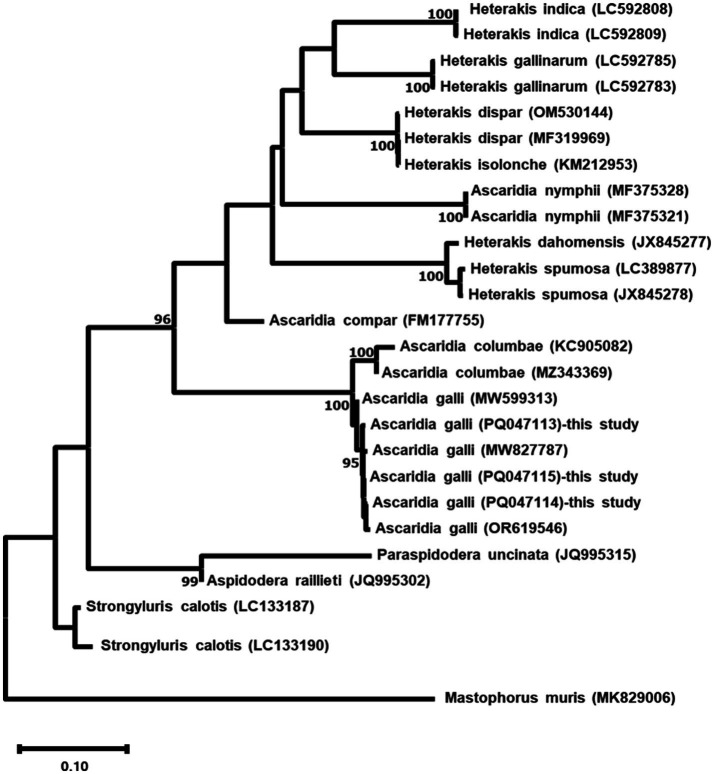
Maximum likelihood phylogenetic tree based on ITS sequences of three *Ascaridia galli* isolates and related nematode species. The tree was constructed using the GTR + G + I model. Numbers at nodes indicate bootstrap support values (%) from 1,000 replicates; only values >90% are shown. *Mastophorus muris* was used as an outgroup. Scale bar represents 0.10 nucleotide substitutions per site.

**Figure 6:**
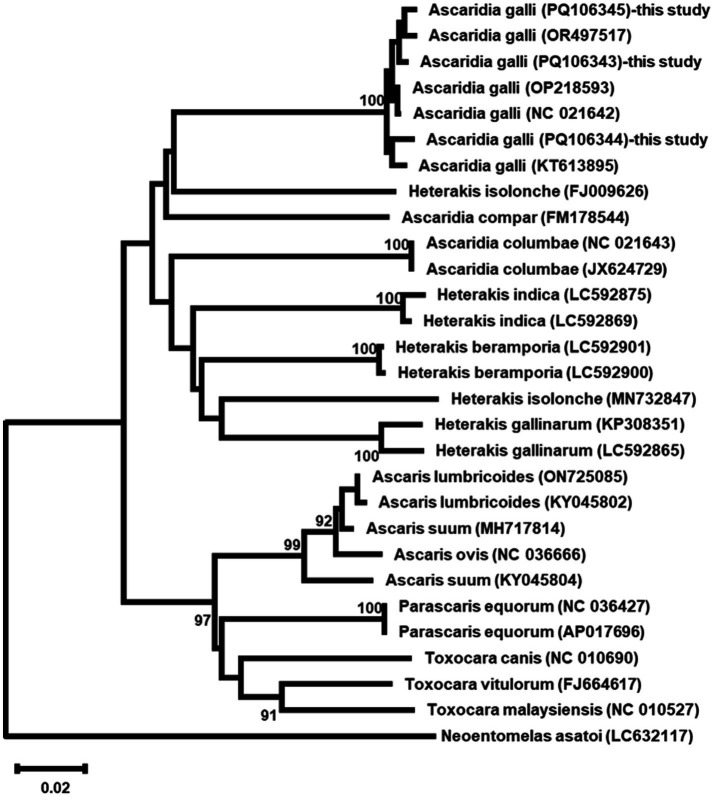
Phylogenetic maximum likelihood tree of COX1 sequences from three *Ascaridia galli* isolates and other related taxa. The tree was constructed using the GTR + G + I model. Numbers at nodes indicate bootstrap support values (%) from 1,000 replicates; only values >90% are shown. *Neoentomelas asatoi* was used as an outgroup. Scale bar represents 0.02 nucleotide substitutions per site.

Phylogenetic analysis based on ITS sequences revealed the three *A. galli* isolates formed a well-supported monophyletic group with 100% bootstrap support, clustering closely with other *A. galli* sequences (Figure 5). COX1 phylogenetic analysis showed identical patterns with the three isolates forming a monophyletic clade (100% bootstrap support) distinct from other *Ascaridia* and *Heterakis* species (Figure 6).

#### ITS and 18S rDNA sequencing of *Eimeria* sp.

Table 2 illustrates that the ITS rDNA region was effectively amplified from three *Eimeria* sp. samples, yielding sequences of 925, 923, and 923 base pairs, with given accession numbers PQ047140, PQ047141, and PQ047142, respectively. The 18S rDNA was successfully amplified from the same three isolates, yielding sequences of 1,754, 1,753, and 1,754 base pairs, deposited under accession numbers PQ142692, PQ142693, and PQ142694, respectively. Sequence analysis confirmed taxonomic identification as *E. tenella*.

**Table 2:** Molecular characterization summary of *Eimeria tenella* isolates.

Marker	Length (bp)	Accession numbers	Intraspecific similarity	Similarity with other *E. tenella*	Key interspecific similarity
ITS	923–925	PQ047140–PQ047142	98.59–98.81%	98.44–99.24%	*E. necatrix*: 75.81–76.99%
18S rDNA	1,753–1,754	PQ142692–PQ142694	99.60–99.66%	98.86–99.83%	*E. necatrix*: 98.80%

Specific intraspecific variations included: ITS sequence PQ047140 differed from PQ047141 by 10 base pair substitutions and 2 gaps, while exhibiting 9 base pair differences and 2 gaps compared to PQ047142. The 18S rDNA sequence PQ142692 showed 6 base pair substitutions compared to PQ142693, and 7 base pair differences relative to PQ142694. Interspecific comparisons showed lower similarities with other *Eimeria* species*: E. gallopavonis* (97.83% for 18S rDNA), and other *Eimeria* species (93.47–97.82%).

#### Phylogenetic relationships

**Figure 7:**
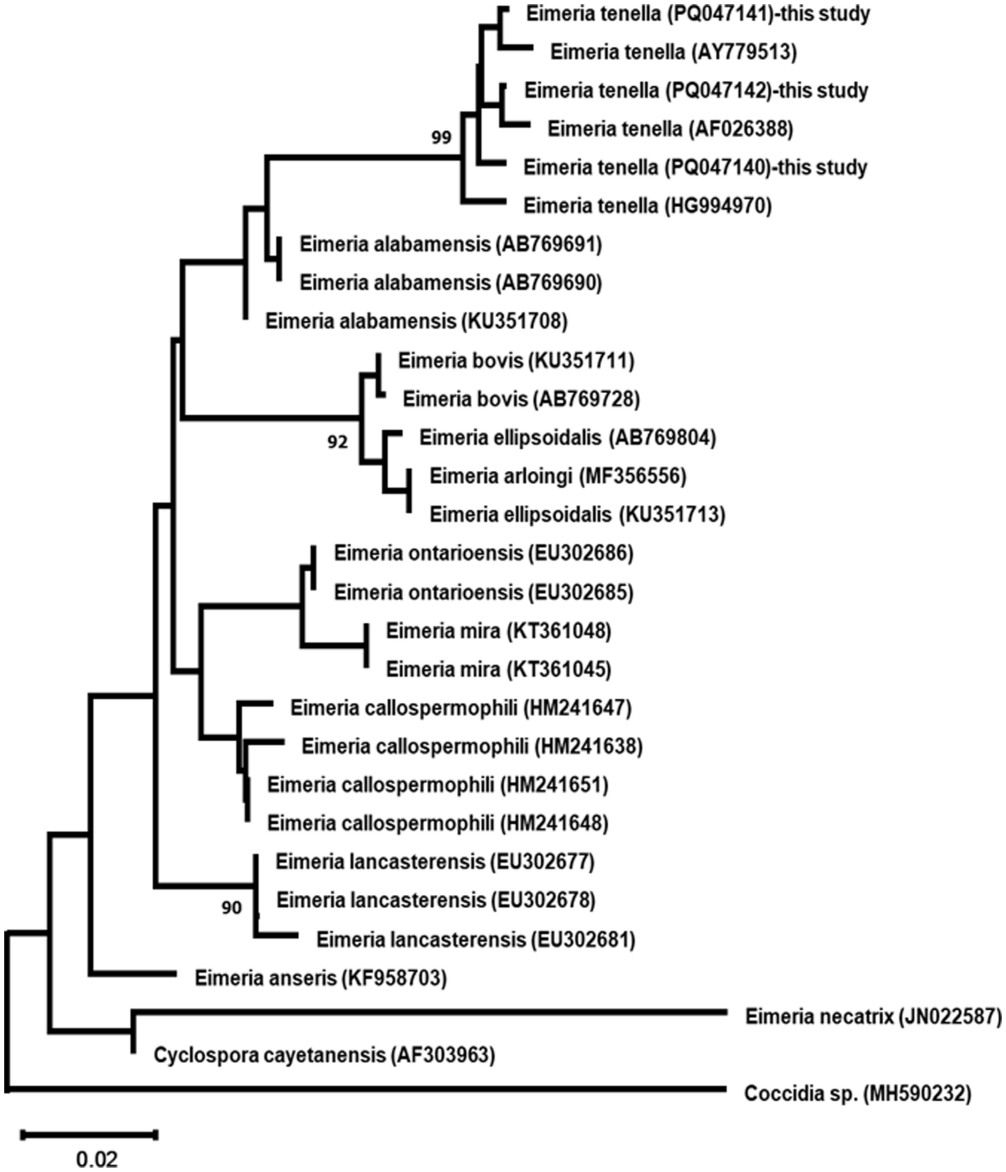
Maximum likelihood phylogenetic tree based on ITS region sequences from three *Eimeria tenella* isolates and other related taxa. The tree was constructed using the GTR + G + I model. Numbers at nodes indicate bootstrap support values (%) from 1,000 replicates; only values >90% are shown. *Coccidia* sp. serves as an outgroup. Scale bar represents 0.02 nucleotide substitutions per site.

**Figure 8:**
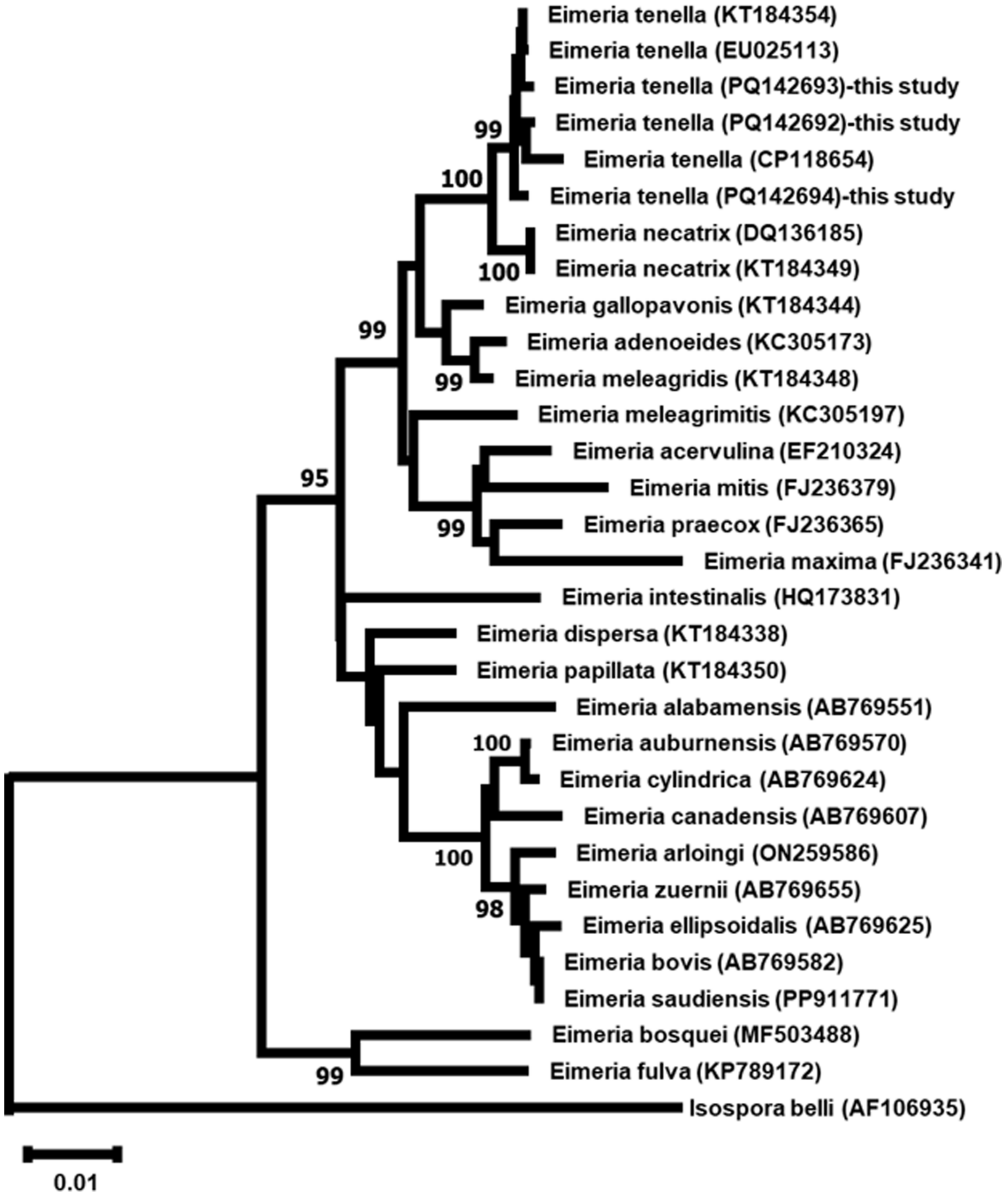
Maximum Likelihood phylogenetic tree based on 18S rDNA sequences from three *Eimeria tenella* isolates and other related taxa. The tree was constructed using the GTR + G + I model. Numbers at nodes indicate bootstrap support values (%) from 1,000 replicates; only values >90% are shown. *Isospore belli* serves as outgroups. Scale bar represents 0.01 nucleotide substitutions per site.

Maximum Likelihood analysis of both ITS and 18S rDNA sequences revealed the three *E. tenella* isolates formed well-supported monophyletic groups (99% bootstrap support), clustering with other *E. tenella* sequences while maintaining clear separation from other *Eimeria* species (Figures 7, 8).

### Histopathological findings

**Figure 9:**
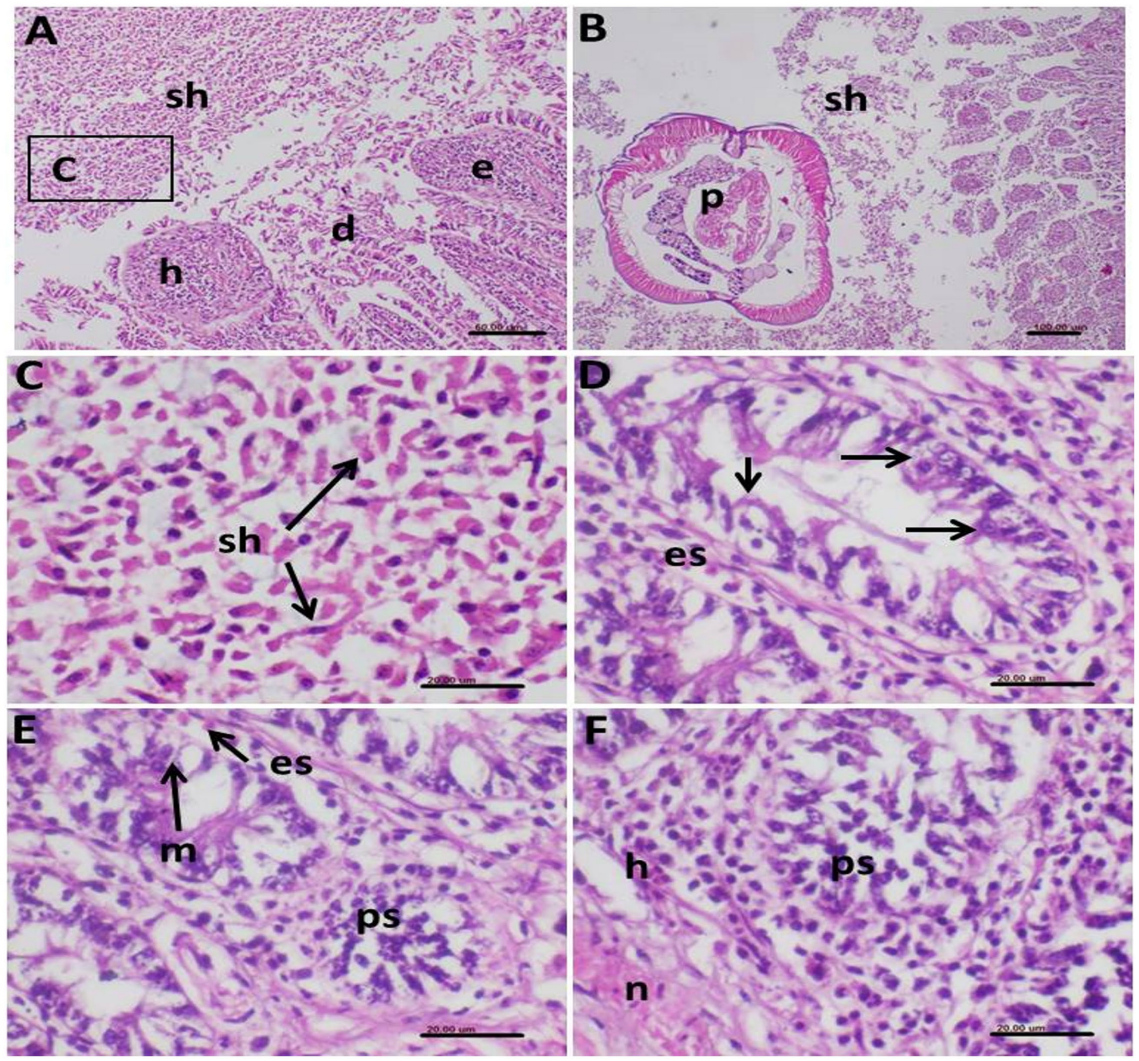
Photomicrograph of histopathological section of the intestine of infected birds with *Eimeria tenella* and stained with hematoxylin and eosin showing **(A)** very extensive infection with many schizonts (sh) in the intestinal lumen associated with hemorrhages (h) and desquamated (d) epithelium (e); **(B)** Some parts of the intestine showed concomitant infection of the shizonts (sh) and nematodes parasites (p); **(C)** Higher magnification of the shizonts of **(A)** demonstrating its characteristic elliptical shape of the schizonts dispersed in remnants of the desquamated epithelial cells; **(D)** Intestinal mucosal epithelium showing intracellular stages of *E. tenella* (arrows) with infiltration with eosinophils in the surrounding tissue; **(E)** Macrogametes (m) of the parasite and other parasite stages (ps) in the epithelium together with eosinophils (es) infiltration; **(F)** Infected mucosal cells showing stages of the *E. tenella* parasite (ps), hemorrhages (h) and necrosis (n).

Histopathological examination of infected birds revealed severe pathological lesions in the intestine. Various stages of *Eimeria* parasites were observed in the epithelial cells of the intestinal mucosa and within the intestinal lumen. In some instances, parasites were aggregated in the lamina propria. Extensive infection with numerous schizonts was noted in the intestinal lumen, associated with hemorrhages and epithelial desquamation (Figure 9A). Concurrent infection with nematode parasites (*A. galli*) was evident, with cross-sections of nematode worms observed alongside dispersed schizonts among remnants of desquamated epithelial cells (Figures 9B,C).

The intestinal mucosal epithelium exhibited intracellular stages of *E. tenella*, accompanied by eosinophilic infiltration in the surrounding tissue (Figure 9D). Macrogametes and other parasite stages were also observed in the epithelium, along with eosinophil infiltration (Figure 9E). Hemorrhage and necrosis were consistently observed in areas of infected mucosal cells (Figure 9F).

**Figure 10:**
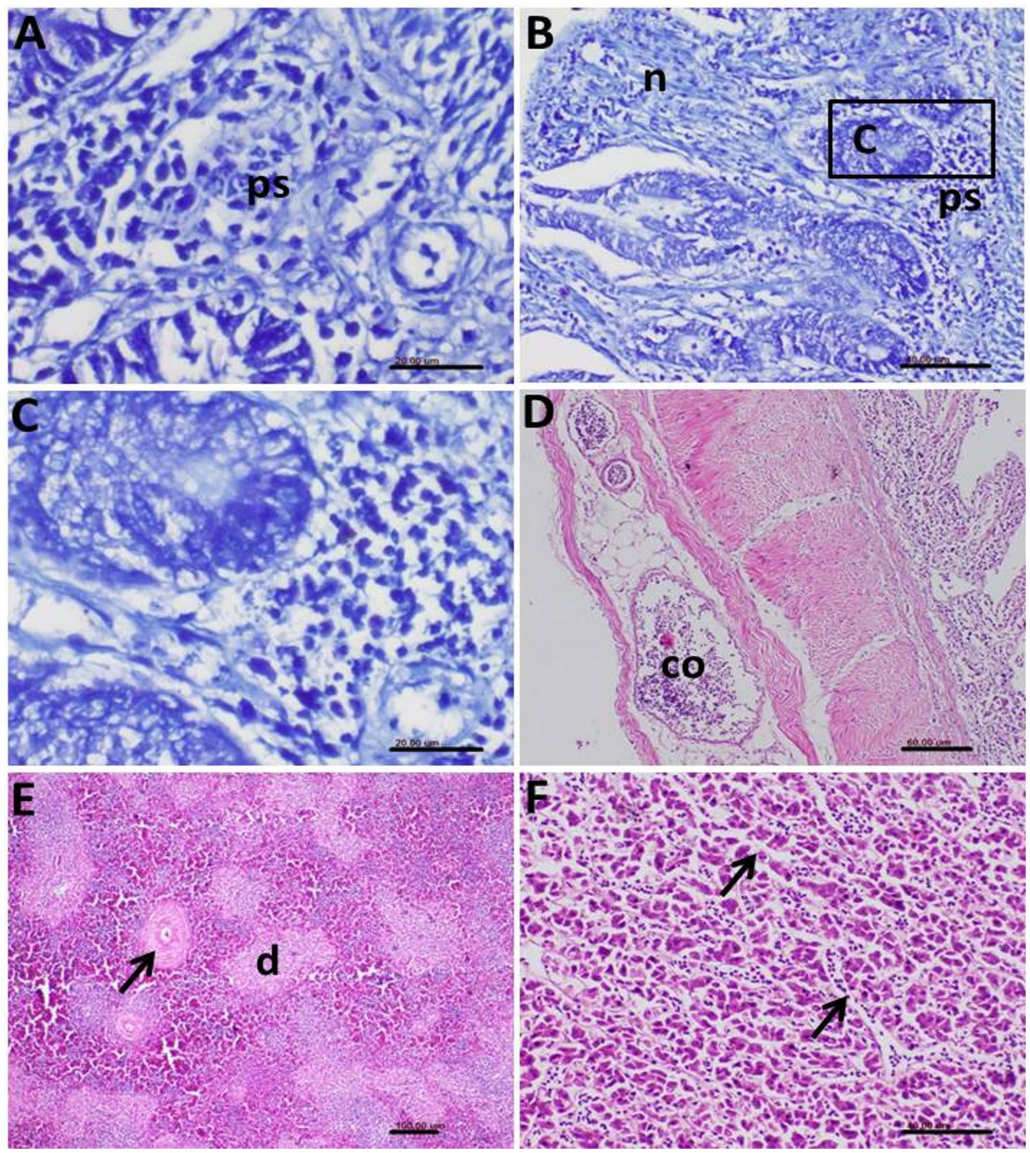
Photomicrograph of histopathological section of the intestine of infected birds with *Eimeria tenella* showing **(A)** Positively stained *Eimeria* stages (ps) by Giemsa stain; **(B)** Giemsa-stained parasite stages (ps) and prominent necrosis (n) of the intestinal epithelium; **(C)** Higher magnification of the previous photo showing many stages of the *Eimeria* and hyperactivity of the mucous glands; **(D)** Congestion (co) of the blood vessels and edema in the serosal layer of the infected intestine; **(E)** Spleen of the infected bird showing depletion (d) of the lymphocytes and thickening in the wall of the follicular arterioles (arrow); **(F)** Liver of the infected birds showing congestion of the hepatic sinusoids (arrows).

*E. tenella* stages in the infected intestinal mucosa showed a positive reaction to Giemsa staining (Figures 10A–C). Some sections of the intestine displayed minor pathological changes, primarily congestion of blood vessels and oedema in the serosal layer (Figure 10D). Secondary pathological changes were noted in other internal organs of the infected birds. The spleen exhibited a depletion of lymphocytes in splenic follicles and a thickening of follicular arteriole walls (Figure 10E).

In the liver, congestion of hepatic sinusoids and multifocal areas of hepatocellular degeneration were common findings (Figure 10F). The severity and extent of these histopathological lesions correlated strongly with the clinical signs and gross pathological findings observed in the affected birds, providing clear evidence of the significant impact of the parasitic infections on the health of the helmeted guineafowl.

## Discussion

The poultry industry is one of the fastest-growing sectors in global animal agriculture, playing a critical role in food security and economic development (52–54). Parasitic diseases remain a significant constraint to poultry health and production worldwide (11, 12). They contribute to substantial economic losses through reduced growth rates, poor feed conversion, decreased egg production, and increased mortality (55).

Parasitism is ubiquitous in wild birds, with individual birds typically hosting a diverse array of parasites throughout their lives (33, 56, 57). However, studying disease in free-ranging species presents unique challenges compared to research in humans or domestic birds (54, 58). These challenges include insufficient baseline data for host species, limited understanding of avian life cycle features, and difficulties in measuring disease-related variables (59). These limitations often hinder the calculation of fundamental epidemiological metrics such as prevalence, incidence, morbidity, and mortality rates, which are crucial for evaluating the impact of parasites on bird populations (57, 60). In the context of domestic poultry, parasitic co-infections pose a significant threat to the chicken industry, potentially causing substantial economic losses (41, 61).

Our study revealed that co-infection with *A. galli* and *E. tenella* resulted in a 10% mortality rate in the flock studied. This finding differs from that reported by Mousa et al. (41), who observed variable mortalities due to the parasitic coinfections in broiler and layer chickens in their study. The disparity in mortality rates could be attributed to differences in bird management practices, environmental conditions, or the genetic background of the chickens, highlighting the need for further research to elucidate factors influencing the severity of co-infections. *A. galli* may cause physical disruption to the intestinal wall and decrease immunity which predispose to *E. tenella*.

The parasites identified in our study, *A. galli* and *E. tenella*, are among the most common gastrointestinal parasites found in the avian gut, along with various cestodes and other *Eimeria* species (11, 12, 29, 62–64). These parasites have the capacity to modulate host immune responses; upregulating genes associated with cell-mediated immunity and stimulating humoral immune receptors. This immunomodulation can lead to the onset of mucosal immunity and various developmental stages of the parasites, which are typically observed during histopathological examination of affected tissues (65–68).

Our morphological analysis of *A. galli* eggs found in infected chicken excreta revealed oval, smooth-shelled eggs measuring 75–96 by 48–59 μm. These findings are consistent with those reported by Mousa et al. (41), who described un-embryonated, ellipsoidal eggs with thick, smooth outer shells measuring 65 to 85 × 49 to 55 μm. The slight variations in size ranges between studies may be due to differences in environmental conditions or host factors affecting parasite development.

The *E. tenella* oocysts identified in our study were characterized by their ovoid shape and double-layered wall, with sporulated oocysts measuring 25 (21–27) μm in length and 18 (15–29) μm in breadth. These dimensions are in close agreement with those reported by Mohamed et al. (69), Mares et al. (70), and Nana-Mariam et al. (71), further confirming the accuracy of our species’ identification.

The molecular characterization of *A. galli* and *E. tenella* isolates from wild birds in this study provides valuable insights into the genetic diversity and phylogenetic relationships of these parasites in natural ecosystems. The successful amplification and sequencing of both the ITS rDNA and COX1 regions for *A. galli* and the ITS and 18S rDNA regions for *E. tenella* allowed for a comprehensive genetic analysis of these important avian parasites in non-domestic settings.

For *A. galli*, the ITS rDNA sequences (PQ047113, PQ047114, PQ047115) and COX1 sequences (PQ106343, PQ106344, PQ106345) demonstrated high similarity to previously reported *A. galli* sequences, confirming their identity in wild bird populations. The observed intraspecific variations, though minor, highlight the genetic diversity within *A. galli* populations in natural environments. This diversity could be attributed to factors such as geographical isolation, adaptation to different wild bird species, or natural genetic drift (37, 72). The genetic variation observed in these wild isolates may differ from that seen in domestic poultry, potentially reflecting adaptations to diverse wild hosts or environmental conditions.

Phylogenetic analysis of both ITS and COX1 sequences consistently placed our *A. galli* isolates from wild birds within a well-supported monophyletic clade, closely related to other *A. galli* sequences. This robust clustering confirms the utility of these genetic markers for species identification across different host species and environments. The clear separation between *Ascaridia* and other genera like *Heterakis* in the phylogenetic trees underscores the effectiveness of these markers in resolving taxonomic relationships at both the genus and species levels, even in diverse wild bird populations.

For *E. tenella*, the ITS rDNA sequences (PQ047140, PQ047141, PQ047142) and 18S rDNA sequences (PQ142692, PQ142693, PQ142694) also demonstrated high similarity to known *E. tenella* sequences, confirming their identity in wild birds. The slight intraspecific variations observed suggest some level of genetic diversity within the *E. tenella* population studied. This diversity could be particularly important in wild bird populations, where parasite transmission dynamics and host–parasite interactions may differ significantly from those in domestic settings (73).

The phylogenetic analyses of both ITS and 18S rDNA sequences placed our *E. tenella* isolates from wild birds in well-supported clades with other *E. tenella* sequences. Comparison with global strains shows close relationships with domestic isolates but reveals subtle differentiation suggesting host adaptation to wild bird transmission cycles. The close phylogenetic relationships observed between our wild bird isolates and those from domestic poultry suggest that *E. tenella* may have a broad host range or that there might be frequent transmission between wild and domestic bird populations. This finding has important implications for the epidemiology and control of coccidiosis in both wild and domestic birds (74).

The molecular analysis revealed significant interspecific divergence between *E. tenella* and other *Eimeria* spp., demonstrating the differential discriminatory power of the two molecular markers used. For 18S rDNA, sequence similarities with other *Eimeria* spp. ranged from 93.47 to 97.82%, with the closest relationship observed with *E. necatrix* (98.80% similarity) and *E. gallopavonis* (97.83% similarity). In contrast, ITS rDNA showed much greater interspecific divergence, with *E. necatrix* similarities ranging only from 75.81 to 76.99%, highlighting the superior discriminatory power of ITS regions for species differentiation. These divergence patterns reflect the evolutionary constraints on different genomic regions, where the highly conserved 18S rDNA maintains higher similarities between species, while the more variable ITS regions better capture species-specific evolutionary signatures. The interspecific distance patterns exceed established taxonomic thresholds for *Eimeria* species boundaries (>2–3% for 18S rDNA, >5% for ITS), confirming robust molecular species delineation (75).

The consistent clustering of *E. tenella* sequences away from other species validates the complementary use of both ribosomal markers for accurate species identification and supports current taxonomic classifications (76). This is particularly important when studying parasites in diverse wild bird communities, where multiple *Eimeria* species may co-exist (77, 78).

Histopathological examination revealed severe pathological lesions caused by *E. tenella* in the intestinal mucosa, particularly in the colon. The observed lesions, including the presence of various developmental stages of the parasite in epithelial cells and the lamina propria, extensive hemorrhages, and desquamation of the epithelium, are consistent with the findings of Mohamed et al. (69) and Salem et al. (12). The concurrent presence of nematode parasites (*A. galli*) alongside *E. tenella* schizonts in some intestinal sections indicates the complexity of the co-infection and its potential for exacerbating tissue damage.

The histopathological changes observed in our study, including necrosis of intestinal mucosa, desquamated epithelial tissue in the intestinal lumen, and inflammatory reaction in the mucosa and submucosa, were closely parallel with the previous reports. These findings highlight the significant impact of parasitic co-infections on intestinal health and function, which likely contribute to the observed clinical signs and mortality. The presence of both *A. galli* and *E. tenella* in the same host raises important questions about potential interactions between these parasites. The co-occurrence of both parasites in histological sections suggests the possibility of synergistic or antagonistic effects. Future research should focus on elucidating the nature of these interactions and their implications for host health and parasite control strategies.

## Conclusion

The current study provides valuable insights into the impact of *A. galli* and *E. tenella* co-infection in helmeted guineafowl. The observed mortality rate, coupled with the severe histopathological changes, underscores the significant threat posed by these parasites to poultry health and production. Future studies should focus on investigating potential interactions between *A. galli* and *E. tenella*, as well as exploring breed-specific susceptibilities to these parasites. Such research will be crucial for developing more effective strategies for preventing and controlling parasitic infections in poultry, ultimately contributing to improved bird health and economic outcomes in the poultry industry.

In addition, this study confirms the utility of ITS rDNA, COX1, and 18S rDNA as reliable genetic markers for the identification and phylogenetic analysis of *A. galli* and *E. tenella* in wild bird populations. The genetic diversity observed within these parasites underscores the importance of continued molecular surveillance in natural ecosystems to monitor for potential changes in parasite populations that could impact wild bird health or pose risks to domestic poultry. The genetic similarities between our wild bird isolates and those from domestic birds highlight the potential for cross-transmission and the need for integrated approaches to parasite management in both wild and domestic avian populations. Further studies incorporating samples from diverse wild bird species across various geographical locations could further elucidate the genetic structure, host specificity, and evolution of these important avian parasites in natural environments.

We recommend that chicken farmers and field veterinarians do regular fecal screenings and necropsy assessments to promptly identify co-infections, particularly in systems with insufficient biosecurity measures. To address dual-pathogen loads, anthelmintic and anticoccidial management strategies should be integrated rather than species-specific. This study provides valuable insights for enhancing guineafowl parasite management strategies and lays a foundation for future research on multi-parasite infections in other avian species.

## Data Availability

The raw data supporting the conclusions of this article will be made available by the authors, without undue reservation.
